# Dental floss–assisted detachable loop ligation followed by snare resection of a giant pedunculated duodenal adenoma

**DOI:** 10.1055/a-2953-9017

**Published:** 2026-09-30

**Authors:** Zhongshang Sun, Lei Xu, Feng Pan

**Affiliations:** 1 Department of GastroenterologyThe Affiliated Huaian No.1 People's Hospital of Nanjing Medical UniversityHuai'anJiangsuChina

A giant pedunculated adenoma, measuring approximately 5.0×3.5 cm ( Fig. 1a ), was identified at the junction between the duodenal bulb and the descending duodenum and was classified as Paris type 0-Ip. 1​ Owing to its large size, the lesion had largely prolapsed into the descending duodenum. Its markedly lobulated surface, bulky head, thick stalk, and occupation of the intestinal lumen impaired endoscopic visualization and made conventional snare placement at the stalk base technically challenging.

**Fig. 1:**
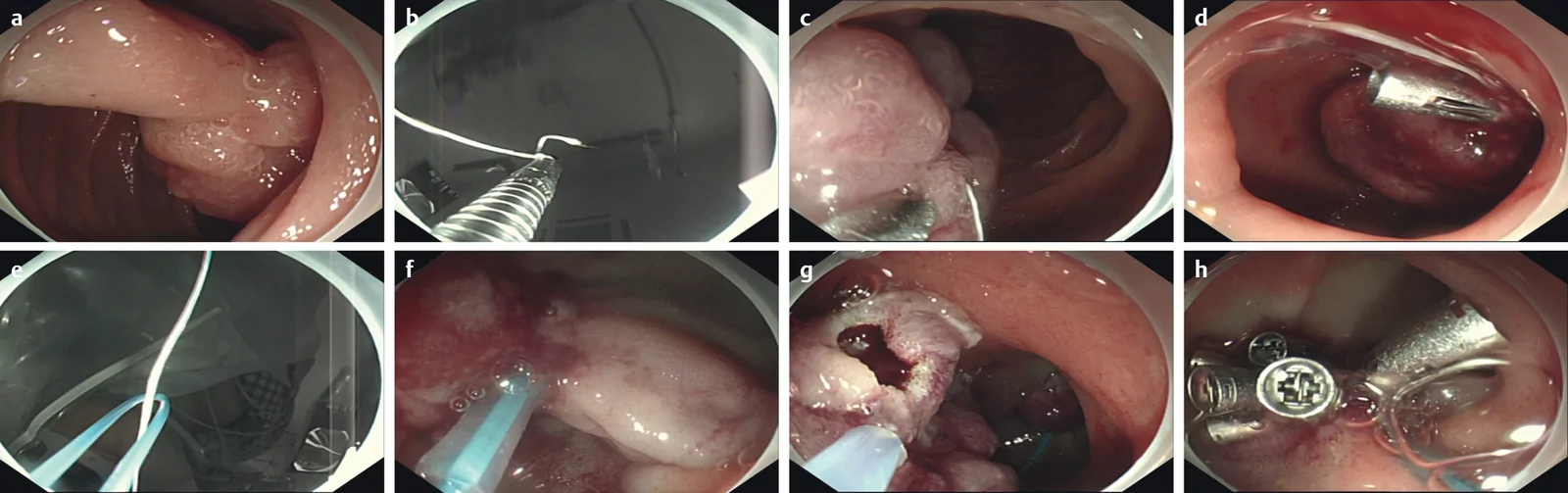
Endoscopic snare resection of a giant pedunculated duodenal adenoma after dental floss–assisted detachable loop ligation. ( **a** ) A giant pedunculated adenoma was identified at the junction between the duodenal bulb and the descending duodenum. ( **b** ) A traction system was created by tying dental floss to the arms of an endoclip. ( **c** ) The endoclip was applied to the head of the adenoma to enable traction. ( **d** ) Most of the adenoma was retracted into the duodenal bulb. ( **e** ) A partially released detachable nylon loop was threaded onto the dental floss and advanced along it toward the duodenal bulb. ( **f** ) The nylon loop was securely tightened around the base of the adenoma stalk for ligation. ( **g** ) The adenoma was completely resected using a conventional snare. ( **h** ) The mucosal defect was completely closed with multiple endoclips.

To overcome this technical challenge and reduce the risk of procedure-related bleeding, we used an external traction-assisted method combined with prophylactic ligation ( Video 1 ). A dental floss line was attached to the adenoma using an endoclip, allowing the main body of the lesion to be pulled back toward the duodenal bulb and exposing the thick stalk ( Fig. 1b–d ). A detachable nylon loop was then loosened outside the patient and passed over the dental floss ( Fig. 1e ). Under continuous traction, the loop was advanced along the floss and positioned securely around the base of the stalk ( Fig. 1f ).

**Video 1** Stepwise endoscopic management of a giant pedunculated duodenal adenoma using dental floss–assisted detachable loop ligation.


After the detachable loop was sufficiently tightened to ligate the feeding vessels within the stalk, the adenoma was completely resected using a conventional snare ( Fig. 1g ). The mucosal defect was subsequently closed with multiple endoclips ( Fig. 1h ). No immediate bleeding or perforation occurred. The patient recovered uneventfully after the procedure, without evidence of delayed hemorrhage or other adverse events.

This case demonstrates that dental floss–assisted traction combined with detachable loop ligation can facilitate safe snare resection of a giant pedunculated duodenal adenoma with a thick stalk, particularly when conventional snare placement is technically difficult and the bleeding risk is high.

Endoscopy_UCTN_Code_TTT_1AO_2AG

## Declaration of GenAI Use

The authors confirm that no generative artificial intelligence was used in the preparation of this manuscript.
